# A framework for incorporating 3D hyperelastic vascular wall models in 1D blood flow simulations

**DOI:** 10.1007/s10237-021-01437-5

**Published:** 2021-03-08

**Authors:** Alberto Coccarelli, Jason M. Carson, Ankush Aggarwal, Sanjay Pant

**Affiliations:** 1 Zienkiewicz Centre for Computational Engineering, College of Engineering, Swansea University, Swansea, UK; 2 Data Science Building, Swansea University Medical School, Swansea University, Swansea, UK; 3 HDR-UK Wales and Northern Ireland, Health Data Research UK, London, UK; 4 Glasgow Computational Engineering Centre, James Watt School of Engineering, University of Glasgow, Glasgow, UK

**Keywords:** Common carotid artery, Hyperelasticity, Tube law, Axial stretching, Pulse wave velocity, One-dimensional blood flow modelling

## Abstract

We present a novel framework for investigating the role of vascular structure on arterial haemodynamics in large vessels, with a special focus on the human common carotid artery (CCA). The analysis is carried out by adopting a three-dimensional (3D) derived, fibre-reinforced, hyperelastic structural model, which is coupled with an axisymmetric, reduced order model describing blood flow. The vessel transmural pressure and lumen area are related via a Holzapfel–Ogden type of law, and the residual stresses along the thickness and length of the vessel are also accounted for. After a structural characterization of the adopted hyperelastic model, we investigate the link underlying the vascular wall response and blood-flow dynamics by comparing the proposed framework results against a popular tube law. The comparison shows that the behaviour of the model can be captured by the simpler linear surrogate only if a representative value of compliance is applied. Sobol’s multi-variable sensitivity analysis is then carried out in order to identify the extent to which the structural parameters have an impact on the CCA haemodynamics. In this case, the local pulse wave velocity (PWV) is used as index for representing the arterial transmission capacity of blood pressure waveforms. The sensitivity analysis suggests that some geometrical factors, such as the *stress-free* inner radius and opening angle, play a major role on the system’s haemodynamics. Subsequently, we quantified the differences in haemodynamic variables obtained from different virtual CCAs, tube laws and flow conditions. Although each artery presents a distinct vascular response, the differences obtained across different flow regimes are not significant. As expected, the linear tube law is unable to accurately capture all the haemodynamic features characterizing the current model. The findings from the sensitivity analysis are further confirmed by investigating the axial stretching effect on the CCA fluid dynamics. This factor does not seem to alter the pressure and flow waveforms. On the contrary, it is shown that, for an axially stretched vessel, the vascular wall exhibits an attenuation in absolute distension and an increase in circumferential stress, corroborating the findings of previous studies. This analysis shows that the new model offers a good balance between computational complexity and physics captured, making it an ideal framework for studies aiming to investigate the profound link between vascular mechanobiology and blood flow.

## Introduction

One-dimensional (1D) network modelling has become an established tool for computing haemodynamic quantities through blood vessels for a broad spectrum of patho-physiological and post-interventional scenarios (Alastruey et al. 2007; Müller and Toro 2014a, b; Mynard and Smolich 2015; Coccarelli et al. 2016; Boileau et al. 2018; Blanco et al. 2017; Sazonov et al. 2017; Coccarelli et al. 2017, 2018a; Carson et al. 2019; Coccarelli et al. 2019; Charlton et al. 2019). The advantage of 1D modelling approach over the more traditional three-dimensional computational fluid dynamics (CFD) and fluid–structure interaction (FSI) models resides in its extremely competitive computational efficiency with an acceptable reduction in accuracy (Alastruey et al. 2016). The gain in computational efficiency with 1D network models is even more notable when comparing with FSI models; both account for the coupling between vascular wall and blood flow, whereas CFD models do not.

One major challenge for realizing a reliable computational haemodynamic solver involves the correct biomechanical characterization of the vascular wall in a patient, which cannot be directly measured in-vivo. For instance, the evaluation of the distensibility of an artery is a cumbersome task due to the intrinsic difficulties in measuring simultaneously diameter and pressure variations at the same point of the vessel. Due to the hierarchical organization of the arterial tree, each blood vessel exhibits unique structural behaviour in order to sustain its specific physiological fluid load. Large arteries are rich in elastic material, whilst smaller vessels present a stiffer behaviour, which is reflected in the increase in pulse wave velocity from core to peripheral regions. Each vessel structure is generally very complex as it is arranged in layers performing different functions and constituted by several components, such as endothelial cells, elastin, collagen, smooth muscle cells and connective tissue. The macroscopic wall mechanical response depends on how these tissue components are arranged within each layer. Collagen is a protein that is able to confer exceptional strength, toughness and mechanical stability to the tissue as it is able to re-arrange its hierarchical structure. With respect to collagen, elastin is much more flexible, allowing the vessel to sustain larger deformation and stress. By isolating the elastin and collagen contributions, Roach and Burton (Roach and Burton 2013) found that for low tension conditions the wall response is dominated by elastin, whilst for high stress, the tissue exhibits the characteristic stiffening of collagen. This is reflected by a stress–strain relationship which is highly nonlinear. The arrangement of the dispersed collagen fibres in both media and adventitia implies a considerable anisotropy of the material. Smooth muscle cells are responsible for the active contractility of the wall and are fundamental for flow regulation by controlling the mechanisms of vasoconstriction and vasodilation. This contractile machinery is predominantly governed by intracellular $\mathrm {Ca}^{2+}$ dynamics and is modulated by the endothelium releasing factors (such as NO, EDH and endothelin) which are able to inhibit or activate specific ionic pathways within the smooth muscle (Chen et al. 2013; Herring and Paterson 2018; Coccarelli et al. 2018b). Due to its high water content, the wall is generally considered quasi-incompressible. Hysteresis phenomena are exhibited when arteries are subjected to cyclic loading, and they are particularly relevant for small calibre arteries (Holzapfel et al. 2000; Gasser et al. 2006). This has been confirmed in the study by Alastruey et al. (2011), where the damping effect due to the wall visco-elasticity was much more significant in distal than in proximal blood vessels. Across the lifetime of an artery, several growth and remodelling processes may occur. These can involve continuous and irreversible changes in the structure. Humphrey et al. (2009) pointed out the existing link between vessel axial stresses and compensatory adaptation mechanisms. Another characteristic of arteries is the presence of residual stresses across the wall, which serve to maintain a fairly uniform stress distribution within the vessel at physiological transmural pressures (Westerhof et al. 2010). Some studies have characterized the axial and circumferential residual stress in aorta and carotid arteries by using measurable quantities, such as the opening angle, and the circumferential and axial curvatures (Delfino et al. 1997; Holzapfel et al. 2007; Sommer et al. 2010; Sommer and Holzapfel 2012). Despite the intrinsic complexity of the system, several advanced computational models have been recently proposed for the study of pathological conditions involving the dysfunction of arterial wall components and potential therapies (Marino et al. 2017; Ferruzzi et al. 2018; Gültekin et al. 2019; Niestrawska et al. 2019; Heusinkveld et al. 2018; Hemmler et al. 2018). In most of the cases, the onset and progression of the vascular disease may depend on both systemic and local haemodynamic conditions. The left ventricle of the heart, indeed, generates pressure and flow pulsations that propagate as fast waves throughout the systemic arterial network. These waves travel from larger vessels of the arterial system, down to the micro-circulation, and are partially reflected as they traverse the vessel network. The travelling speed of these pressure pulses is defined as the pulse wave velocity, which strongly depends on the structural properties of the wall. In clinical practice, the measurement of PWV represents a gold-standard technique for assessing the arterial stiffness, providing a reliable prognostic index for cardiovascular morbidity and mortality (Westenberg et al. 2012; Spronck et al. 2015). Among the different established protocols for evaluating this index, the most common one considers the time delay of a pulse transiting between two locations, which are generally the common carotid and the femoral arteries. PWV is calculated by dividing the distance between the two monitoring sites by the pulse time lag recorded at these locations. The quantity obtained is an indicative measure of the global stiffness of the arterial system. Moreover, this index can also be either directly measured or estimated at the local level of a single vascular bed (Perreira et al. 2015; Engelen et al. 2015). This locally evaluated PWV can therefore inform about the specific stiffness of the vessel. An intriguing aspect regarding arterial stiffness is that this condition may depend not only on the material (constitutive) properties, but also on the presence of residual stress and external (not involving the fluid) loading. All arteries are indeed subjected to axial forces which tightly control the length of the vessel. Any perturbation of these, such as those caused by a surgical intervention, may have a significant impact on the arterial structure and therefore on the systemic blood circulation.

To the authors’ knowledge, there are few haemodynamic studies that consider a complete description of the vascular wall mechanics (Alastruey et al. 2011; Vahedein and Liberson 2019; Bertaglia et al. 2020). In most of the 1D blood modelling works, the link between pressure and area (alternatively radius) of the vessel is expressed through either a linear or a hyperbolic relationship, parameterized to represent only a narrow spectrum of patho-physiological conditions. Furthermore, the derivation of these models does not accounts for the characteristic structural features of the wall, such as nonlinearity, anisotropy and residual stresses. Given the fundamental role of vascular wall in determining the blood flow, a more comprehensive and microstructurally informed representation is warranted. This is likely to be especially relevant for clinical cases where the vascular wall function differs from the normal conditions. Therefore, in this study, we present a new methodology for computing blood flow in arterial blood vessels, which incorporates the available biomechanical and microstructural information regarding vascular wall. This is then followed by a rigorous analysis of the effects of the wall structural features on the haemodynamic variables.

## Proposed model

Here the modelling methodology for describing the CCA haemodynamics is described. This consists of a 1D fluid dynamics representation for blood flow in compliant vessels and employs a large strain elastic model for describing the passive response of the vascular tissues to pressure loading.

### Blood flow

**Fig. 1:**
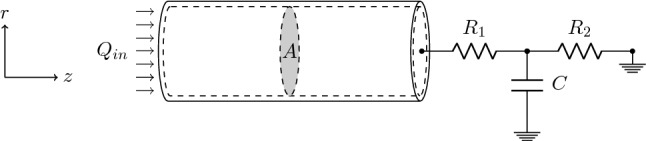
Haemodynamic circuit representing the CCA and its boundary conditions: at the inlet inflow $Q_{in}$=$Q_{in}(t)$ is prescribed, whilst outflow conditions are modelled by a three element ($R_1$,*C*,$R_2$) Windkessel model

The flow domain, the lumen of a large artery, is considered to be of cylindrical shape with axial direction along coordinate *z* and lumen (internal cross-section) area *A* at any time *t* (Fig. 1). Blood flow is approximated to be laminar, Newtonian and axisymmetric. That is, the radial and circumferential components of velocities are much smaller than the axial component. Furthermore, blood is considered to be an incompressible fluid. If the average blood pressure in the lumen *P* and the volumetric blood flow rate *Q* are considered to be the primary variables, the mass and momentum conservation equations at any axial location can be written as

1 $$\begin{aligned} \begin{aligned} \frac{\partial A}{\partial P}\frac{\partial P}{\partial t}+\frac{\partial Q}{\partial z}&= 0 \text {, and } \\ \quad \frac{\rho }{A}\frac{\partial Q}{\partial t}+\frac{\rho }{A} \frac{\partial }{\partial z}\left( \frac{Q^2}{A}\right) + \frac{\partial P}{\partial z} - \frac{f}{A}&= 0, \end{aligned} \end{aligned}$$

respectively. Here $\rho$ represents blood density and *f* is the frictional force per unit length.

The two conservation equations are closed by defining a constitutive relationship between the lumen area and pressure, which is commonly called the “tube law” in literature. This depends on the type of biomechanical model adopted for representing the vascular wall, and it is treated in Sect. 2.2. We note that the variation in the lumen area with respect to the fluid pressure defines the instantaneous vessel compliance $C_A={\partial A}/{\partial P}$ and can be determined from the constitutive law.

By assuming a Poiseuille velocity profile, it is possible to rewrite the mass and momentum conservation equations as

2 $$\begin{aligned} \begin{aligned} C_A\frac{\partial P}{\partial t}+\frac{\partial Q}{\partial z}&= 0 \text {, and } \\ \quad \frac{\rho }{A}\frac{\partial Q}{\partial t}+\frac{\rho }{A} \frac{\partial }{\partial z}\left( \frac{Q^2}{A}\right) + \frac{\partial P}{\partial z} +8\pi \mu \frac{Q}{A^2}&= 0, \end{aligned} \end{aligned}$$

where $\mu$ is the dynamic viscosity of blood. In order to close the haemodynamic model, it is necessary to define boundary conditions at the inlet and outlet of the fluid domain. As boundary conditions, volumetric flow $Q_{in}(t)$ is prescribed at the inlet of the vessel, whilst the downstream vasculature is represented by means of a three-element Windkessel model (Fig. 1).

The intrinsic vessel wave speed *c* is defined as the speed at which infinitesimally small pulses propagate through an initially stressed tube (i.e. $P(t=0)>0$ everywhere) (Mynard and Nithiarasu 2008). According to Carson (2018), this quantity can be related to the arterial compliance $C_A$ via

3 $$\begin{aligned} c=\sqrt{\frac{A}{\rho }\frac{\partial P}{\partial A}}=\sqrt{\frac{A}{\rho }\frac{1}{C_A}}. \end{aligned}$$

Wall compliance and intrinsic wave speed can be also linked to the vessel distensibility *D*, which represents a metric employed in clinical settings for assessing structural stiffness (Engelen et al. 2015; Spronck and Humphrey 2019). This quantity can be directly computed from *in-vivo* signal as

4 $$\begin{aligned} D=\frac{\varDelta A}{A_d}\frac{1}{\varDelta P}=\frac{A_s-A_d}{A_d}\frac{1}{P_s-P_d}, \end{aligned}$$

in which $P_s$, $P_d$, $A_s$ and $A_d$ are the systolic and diastolic values of pressure and area. From the vessel distensibility it is possible to obtain an indicative measurement of the local PWV via the Bramwell–Hill equation (Engelen et al. 2015; Spronck and Humphrey 2019):

5 $$\begin{aligned} {\text{PWV}}_{BH}=\sqrt{\frac{1}{\rho D}}. \end{aligned}$$

**Fig. 2:**
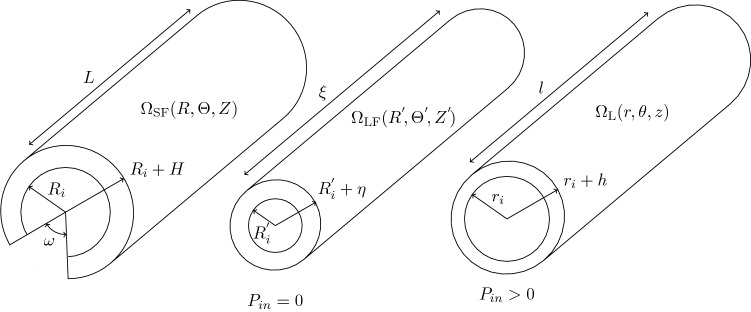
Configurations considered for the system under analysis: *stress-free* $\varOmega _{\text {SF}}$, *load-free* $\varOmega _{\text {LF}}$ and *loaded* $\varOmega _{\text {L}}$

### Vascular mechanics

In the proposed framework, the constitutive relationship between lumen area and luminal blood pressure is derived from a 3D hyperelastic description of the vascular wall. Later, we will make comparisons of this model against the widely used, simpler, tube law (presented in Sect. 3.3).

#### Structure kinematics

Consistent with the flow model, an axisymmetric deformation is assumed for the vascular wall. Since residual strains have been reported in large arteries and shown to play an important role in their mechanical properties, they are incorporated by considering *stress-free*, *load free*, and *loaded* configurations, $\varOmega _{\text {SF}}$, $\varOmega _{\text {LF}}$ and $\varOmega _{\text {L}}$, respectively (Fig. 2). In the *load-free* state, although no external load is applied, there are residual stresses in the vessel wall, whereas in the *stress-free* configuration, the vessel is free from stresses along the radial and axial directions. In a laboratory, the residual stresses are released by cutting the samples along axial and circumferential directions. The residual stresses are generally evaluated from the opening angle $\omega$ and axial shortening that the sample exhibits after the cut.

The three configurations are expressed in the cylindrical coordinates as

6 $$\begin{aligned} \begin{aligned} \varOmega _{\text {SF}}&: R_i \le R\le R_i+H,~~~0\le \varTheta \le (2\pi -\omega ),~~~0\le Z\le L,\\ \varOmega _{\text {LF}}&: R'_i \le R' \le R'_i+\eta ,~~~0\le \varTheta ' \le 2\pi ,~~~~~~~~~~0\le Z'\le \xi , \text { and}\\ \varOmega _{\text {L}}&: r_i \le r \le r_i+h,~~~~~~0\le \theta \le 2\pi ,~~~~~~~~~~~~0\le z\le l. \end{aligned} \end{aligned}$$

Here ($R_i$, *H*, *L*), ($R'_i$, $\eta$, $\xi$), and (*r*_*i*_, *h*, *l*) are the inner radii, the thicknesses and lengths corresponding to the configurations $\varOmega _{\text {SF}}$, $\varOmega _{\text {LF}}$ and $\varOmega _{\text {L}}$, respectively. The lumen area *A* is related to the internal radius in the loaded configuration $A=\pi r_i^2$.

It is assumed that the vessel axial length does not vary with loadings imposed by the fluid flow. That is, $l=\lambda _{z,res}\lambda _{z,ext} L$, where $\lambda _{z,res}$ is the residual axial stretch, whilst $\lambda _{z,ext}$ is the axial stretch due to external loading. The vascular wall is assumed to be incompressible, which implies a mapping

7 $$\begin{aligned} \varOmega _{\text {SF}} \rightarrow \varOmega _{\text {L}}: r=\sqrt{\frac{R^2-R^2_i}{k\lambda _{z}}+r_i^2}. \end{aligned}$$

Here, *k* is a parameter defined as $k=2\pi /(2\pi -\omega )$ and $\lambda _{z}=\lambda _{z,res}\lambda _{z,ext}$ is the net axial stretch. Therefore, in the absence of torsion, the deformation gradient of the mapping from stress-free to the loaded configuration is diagonal and can be written as Holzapfel et al. (2000)

8 $$\begin{aligned} \mathbf {F} = {\mathrm{diag}}\left[ \frac{\partial r}{\partial R}, \frac{r}{R} \frac{\partial \theta }{\partial \varTheta }, \frac{l}{L} \right] = {\mathrm{diag}}\left[ \frac{R}{rk\lambda _z}, \frac{kr}{R}, \lambda _{z} \right] . \end{aligned}$$

Importantly, from (7) and (8), it can be seen that the kinematics everywhere on the artery is fully described in terms of only the internal radius $r_i$ and the fixed parameters describing the opening angle $\omega$ and axial stretch $\lambda _z$. The three principal stretches are along the coordinates

9 $$\begin{aligned} \begin{aligned} \lambda _{r}&=\frac{R}{rk\lambda _z}, \\ \lambda _{\theta }&=\frac{kr}{R},\\ \lambda _{z}&=\lambda _{z,res}\lambda _{z,ext}. \end{aligned} \end{aligned}$$

The right Cauchy–Green strain tensor is $\mathbf {C}=\mathbf {F}^\top \mathbf {F}$ and the first strain invariant $I_1={\mathrm{tr}}\left( \mathbf {C}\right) = \lambda _r^2+\lambda _{\theta }^2+\lambda _{z}^2$. It has been shown that large arteries are reinforced with families of collagen fibres that are helically arranged (Gasser et al. 2006). If a collagen fibre family is at a helical angle $\phi ^i$, the unit vector along the fibre direction in cylindrical coordinates is $\varvec{n}^i = \left[ 0,\cos (\phi ^i),\sin (\phi ^i)\right]$. Then, the fourth strain invariant is $I_4^i=\varvec{n}^i\cdot \mathbf {C}\varvec{n}^i = \lambda _{\theta }^2 \text {cos}^2\phi ^i+\lambda _{z}^2 \text {sin}^2\phi ^i$ (Holzapfel et al. 2000).

#### Equilibrium equations

If the vascular wall is considered to be a hyperelastic material with strain energy density $\varPsi (I_1,I_4^i)$, the Cauchy stress tensor can be derived as $\varvec{\sigma } = 2\mathbf {F}\dfrac{\partial \varPsi }{\partial \mathbf {C}}\mathbf {F}^\top -p\mathbf {I}$, where *p* acts as the Lagrange multiplier to enforce incompressibility (Auricchio et al. 2014). Thus,

10 $$\begin{aligned} {\sigma }_{rr}=\lambda _{r}\frac{\partial \varPsi }{\partial \lambda _{r}}-p, \quad \quad {\sigma }_{\theta \theta }=\lambda _{\theta }\frac{\partial \varPsi }{\partial \lambda _{\theta }}-p, \quad \quad {\sigma }_{zz}=\lambda _{z}\frac{\partial \varPsi }{\partial \lambda _{z}}-p. \end{aligned}$$

It is easy to see that the momentum equilibrium equation in $\theta$ is identically satisfied, whilst that in the axial direction is not required since we have assumed $\lambda _z$ to be fixed (see Appendix A3 for complete equations). Thus, we are left with only the radial component, which can be written as

11 $$\begin{aligned} d\sigma _{rr} = \frac{\sigma _{\theta \theta }-\sigma _{rr}}{r}dr. \end{aligned}$$

Integrating across the thickness and using the boundary conditions that $\sigma _{rr}$ is equal to the applied pressures at the internal and external surfaces (i.e. $\sigma _{rr}(r_i)=-P_{in}$ and $\sigma _{rr}(r_i+h)=-P_{ext}$), we get

12 $$\begin{aligned} P_{in}-P_{ext} = \int \limits _{r_i}^{r_i+h} \frac{{\sigma }_{\theta \theta }-{\sigma }_{rr}}{r}dr, \end{aligned}$$

in which $P_{in}$ and $P_{ext}$ are the pressures acting on the inner and outer surfaces of the wall, respectively. In this study the luminal pressure $P_{in}$ is assumed to equalize the average blood pressure over the cross-sectional area *P* reported in Sect. 2.1 (and therefore *P* replaces $P_{in}$ in the following). On the other hand, the external pressure $P_{ext}$ can be assumed constant along the length of the vessel. By inserting the first two expressions of (10) into (12) yields

13 $$\begin{aligned} P-P_{ext}=\int \limits _{r_i}^{r_i+h} \left( \lambda _{\theta }\frac{\partial \varPsi }{\partial \lambda _{\theta }}-\lambda _{r}\frac{\partial \varPsi }{\partial \lambda _{r}}\right) \frac{1}{r}dr. \end{aligned}$$

Finally, the integration variables are changed from current $\varOmega _{\text {L}}$ to reference $\varOmega _{\text {SF}}$ coordinates in order to obtain

14 $$\begin{aligned} P-P_{ext}=\int \limits _{R_i}^{R_i+H} \left( \lambda _{\theta }\frac{\partial \varPsi }{\partial \lambda _{\theta }}-\lambda _{r}\frac{\partial \varPsi }{\partial \lambda _{r}}\right) \frac{1}{\lambda _{\theta }\lambda _z r}dR. \end{aligned}$$

The axial force can be calculated by integrating the axial stress over the cross-sectional area, which yields (see Appendix A3 for details)

15 $$\begin{aligned} F_{z}=\pi \int \limits _{R_i}^{R_i+H} \left( 2\lambda _{z}\frac{\partial \varPsi }{\partial \lambda _{z}}-\lambda _{\theta }\frac{\partial \varPsi }{\partial \lambda _{\theta }}-\lambda _{r}\frac{\partial \varPsi }{\partial \lambda _{r}}\right) \frac{r}{\lambda _{\theta }\lambda _z}dR. \end{aligned}$$

#### Constitutive model

The CCA is described as a monolayer elastic tube reinforced with two families of collagen fibres. Each fibre direction is identified by an orientation angle $\phi ^i$ with respect to the circumferential direction. In this study, the strain energy function (SEF) $\varPsi$ proposed in Holzapfel et al. (2005) is adopted

16 $$\begin{aligned} \varPsi =k_0(I_1-3)+\frac{k_1}{2 k_2}\sum _{i=1,2}\{ \text {exp}[k_2\{(1-\alpha )(I_1-3)^2+\alpha (I_4^i-1)^2\}]-1\}, \end{aligned}$$

where $k_0$, $k_1$, $k_2$ and $\alpha$ are material parameters. For the adopted constitutive relationship, it is assumed that collagen fibres cannot sustain compression loads. Therefore, the anisotropic contribution $(I_4^i-1)$ is set to zero when $I_4^i<1$. It is important to highlight that, for the proposed framework, the choice in terms of SEF is not limited to (16), but also other constitutive relationships, such as Gasser et al. (2006), Baek et al. (2007), can be employed. Similarly, multiple layers of the arteries with different constitutive models/parameters can be considered.

Finally, combining (14) and (16), the lumen pressure *P* can be expressed as a function of the following parameters

17 $$\begin{aligned} P-P_{ext} = \widehat{P}(r_i,H,R_i,\lambda _z,\omega ,k_0,k_1,k_2,\alpha ,\phi ^i). \end{aligned}$$

Since $r_i=\sqrt{A/\pi }$, the tube law is thus established. For brevity we write the relation as

18 $$\begin{aligned} P-P_{ext} = \bar{P}(A,H,R_i,\lambda _z,\omega ,k_0,k_1,k_2,\alpha ,\phi ^i)= \bar{P}(A,\bar{\psi }), \end{aligned}$$

where $\bar{\psi }$ is a vector containing the parameters, i.e. $\bar{\psi }=\left\{ H,R_i,\lambda _z,\omega ,k_0,k_1,k_2,\alpha ,\phi ^i\right\}$.

## Methods and cases

### Solution procedure for the coupled wall-mechanics and haemodynamics models

The haemodynamic equations (2) accounting for wall mechanics (18) are solved by employing the implicit sub-domain collocation scheme described in Carson and Van Loon (2017). Firstly, the system (2) is discretized in time as follows

19 $$\begin{aligned} \begin{aligned} C_A^n\frac{\partial P}{\partial t}+\frac{\partial Q^{n+1}}{\partial z}&= 0, \\ \frac{\rho }{A^n}\frac{\partial Q}{\partial t} + \frac{\partial P^{n+1}}{\partial z}&=-\Bigg (\frac{\rho }{A}\frac{\partial }{\partial z}\left( \frac{Q^2}{A}\right) +8\pi \mu \frac{Q}{A^2}\Bigg )^n, \end{aligned} \end{aligned}$$

where $n+1$ represents the current time iteration as all the quantities are known at the previous time step *n*. Then, this system is integrated in space (axial direction *z*) by using the trapezoidal rule and discretized in time by employing a second-order backward difference scheme. The discretized system may be re-written at the element level in the following compact form:

20 $$\begin{aligned} \mathbf{F} _e \mathbf{P} _e^{n+1}+\mathbf{G} _e \mathbf{Q} _e^{n+1}=\mathbf{h} _e^{n}, \end{aligned}$$

in which *e* represents the elemental level, $\mathbf{F} _e$, $\mathbf{G} _e$ are the stiffness matrices of pressure and flow, $\mathbf{P} _e^{n+1}$ and $\mathbf{Q} _e^{n+1}$ the vectors containing the current element values of pressure and flow, and $\mathbf{h} _e^n$ the vector representing convection and diffusion components evaluated at the previous time step. Since the constitutive law (18) is highly non-linear with respect to the luminal area, the compliance is calculated, for simplicity, using central finite difference. Thus, since $C_A ={\partial A}/{\partial P} = {1}/{(\partial P}/{\partial A)}$, the compliance is approximated as

21 $$\begin{aligned} C_A \approx \frac{2 \epsilon }{\bar{P}(A+\epsilon ,\bar{\psi })-\bar{P}(A-\epsilon ,\bar{\psi })} \end{aligned}$$

with $\epsilon =10^{-8}$, whilst $\bar{P}$ is numerically computed from (18) by means of Simpson’s integration rule. Note that solving the system of equations (20) provides the values of pressure and flow rate at time step $n+1$. Subsequently, the area $A^{n+1}$ also needs to be calculated by solving (18). Secant method is utilized in order to achieve this. Given pressure $P^{n+1}$ and a current guess of the area $A^{n+1}\approx A^k$, the solution is updated iteratively using the relation

22 $$\begin{aligned} A^{k+1} = A^{k} + \left( P^{n+1} - P_{ext} - \bar{P}(A^{k},\bar{\psi }) \right) {C_A}^k, \end{aligned}$$

until the residual $A^{k+1}-A^k$ is less than $10^{-6}$.

### Reference parameters for CCA and simulation settings

Here all the necessary details concerning the choice of reference parameters for the CCA and the setup of numerical experiments are reported. With regard to the CCA structural characterization, reference works are by Delfino et al. (1997) and Sommer et al. (2010). In the study by Delfino et al. (1997), the intact specimens of CCA were loaded by an internal pressure at three fixed axial stretches. The applied axial force was varied in order to hold the axial stretch constant during the test. Sommer et al. (2010) defined the biaxial mechanical properties of healthy aged non-atherosclerotic human carotid arteries at physiological and supra-physiological loadings conditions. This was done by means of cyclic, quasi-static extension-inflation tests at different axial pre-stretches. In this case, the vascular tissue exhibited a strong non-linear, pseudo-elastic mechanical behaviour with small hysteresis. Auricchio et al. (2014) presented a rigorous study in which the geometrical and constitutive CCA parameters relative to four different strain energy functions (Holzapfel et al. 2000, 2005; Gasser et al. 2006; Baek et al. 2007) were identified by fitting the experimental curves reported in Delfino et al. (1997); Sommer et al. (2010). The geometrical and constitutive parameters corresponding to SEF (16) by Holzapfel et al. (2005) are summarized in Table 1.

**Table 1:** Fitting parameters for CCA SEF (16), reported from Auricchio et al. (2014). In both cases two families of fibres are considered ($n_f$=2)

Experiment	*H* (cm)	$R_i$ (cm)	$\omega$ ($^o$)	$k_0$ (kPa)	$k_1$ (kPa)	$k_2$ (-)	$\alpha$ (-)	$\phi ^i$ ($^o$)
Delfino et al. (1997)	0.09	0.446	100.0	15.9	158.3	9.8	0.0	± 90.0
Sommer et al. (2010)	0.117	0.431	80.9	26.6	20.9	56.5	0.97	± 24.9

The difference in the experimental curves reported in Delfino et al. (1997); Sommer et al. (2010) is reflected in the discrepancy between the corresponding computed fitting parameters (reported in Table 1). It is worth mentioning that the parameters obtained by Auricchio et al. (2014) from the dataset (Sommer et al. 2010) matched well the values reported in Sommer and Holzapfel (2012). In the current study, these values (computed in Auricchio et al. (2014) from Sommer’s dataset) are used in all the following numerical experiments, unless specified otherwise. The external pressure $P_{ext}$ is considered negligible, whilst blood density $\rho$ and dynamic viscosity $\mu$ are set equal to 1.06 g/cm$^3$ and 0.04 poise, respectively. The length of the *stress-free* vessel *L* is assumed to be 9.0 cm, which is divided in 14 equally sized elements of length $l_{e}$.

The time step chosen for each case is adaptive in order to maintain the Courant–Friedrichs–Lewy (CFL) number close to 2.5. The time step for each simulation is adapted by utilizing the wave speed *c* that is calculated from equation (3) as

23 $$\begin{aligned} \varDelta t = 2.5 l_{e}/c. \end{aligned}$$

This is performed in order to be consistent for each simulation and to limit the error that may arise during the calculation of some haemodynamic variables, such as PWV. The flow through the artery is assumed to be under non-pathological resting conditions, unless otherwise stated. For the inflow signal under resting conditions and the Windkessel model coefficients, the works (Figueroa et al. 2006; Xiao et al. 2014) are followed (see Appendix A1). For each simulation case, the solution is recovered after computing 10 consecutive cycles prescribing the same boundary conditions, which ensures periodic convergence of the solution. All simulations were performed in MATLAB R2019b (The MathWorks, Inc, Natick, Massachusetts) by using a in-house code made open-source (Coccarelli et al. 2021).

### Classical tube law and comparison to hyperelastic model

In the current study, the proposed model is compared against one of the most popular choices of tube laws. The latter relates luminal pressure to lumen area via (Olufsen et al. 2000; Formaggia et al. 1999; Mynard and Nithiarasu 2008)

24 $$\begin{aligned} P=P_{ref}+\beta \left( \sqrt{A}-\sqrt{A_{ref}} \right) , \end{aligned}$$

where $P_{ref}$ is a reference pressure for *A*=$A_{ref}$ and $\beta$ is a parameter expressing the vessel elasticity. It is worth noticing that the expression (24) is linear with respect to the luminal radius $r_i$, and therefore, it is referred as “linear” model. For this tube law, an expression for the compliance can be obtained by differentiating (24) with respect to *A*:

25 $$\begin{aligned} C_A=\bigg (\frac{\partial P}{\partial A}\bigg )^{-1}=\frac{2 \sqrt{A}}{\beta }. \end{aligned}$$

In order to make a meaningful comparison, it is necessary setting a $\beta$ value able to provide a linearized counterpart of the proposed model. Here, it is assumed that the “linear” model has the same compliance as the current “non-linear” model at the diastolic cross-sectional area $A_d$ under resting flow conditions. This latter value corresponds to the minimum cross-sectional area recorded during the cardiac cycle. Therefore, by re-arranging (25), it is possible to calculate the corresponding $\beta _d$ as

26 $$\begin{aligned} \beta _d={\frac{2\sqrt{A_d}}{C_{A,d}}}, \end{aligned}$$

where $C_{A,d}$ is the compliance at diastole, which is evaluated from the current hyperelastic model. Since the variation in compliance with respect to *A* is significant, one more case, accounting for an averaged value of $C_A$ over the systolic-diastolic *A* range ($C_{A,a}$), is considered. For this other “linear” model, the corresponding $\beta _a$ is evaluated as

27 $$\begin{aligned} \beta _a={\frac{2\sqrt{A_d}}{C_{A,a}}}. \end{aligned}$$

It is worth mentioning that both $\beta _{d}$ and $\beta _{a}$ are calculated after computing the solution for the current hyperelastic model. Although each of these parameters is constant, the compliance of the “linear” model varies with *A*, according to (25).

## Results and discussion

The proposed theoretical framework is employed for analysing the underlying link between the structural function of the wall and the haemodynamics in the human CCA. This section is structured as follows. In the first part, the response of the considered hyperelastic wall model to biaxial testing conditions is analysed. From these numerical results, a physiologically relevant *in-vivo* stress is identified and adopted in following simulations. Then, the proposed vascular model is compared against a standard tube law. The characterization of the haemodynamic response is carried out by considering characteristic haemodynamic quantities such as the local PWV, the pressure and area at diastole and systole. Through this qualitative comparison, the characterizing features of the model are presented. Then, we report a multivariate sensitivity analysis aiming to define the impact of each wall structural parameter on the local PWV. This analysis aims to identify the vascular components which play a major role in the blood–wall interaction. Once the key model parameters are identified, we compare the performances of the tube laws by considering three different CCAs (with most relevant parameters varying) and two flow conditions (to simulate a change in physiological state). Finally, the effects axial stretching on the CCA blood dynamics are investigated.

### Hyperelastic wall behaviour

**Fig. 3:**
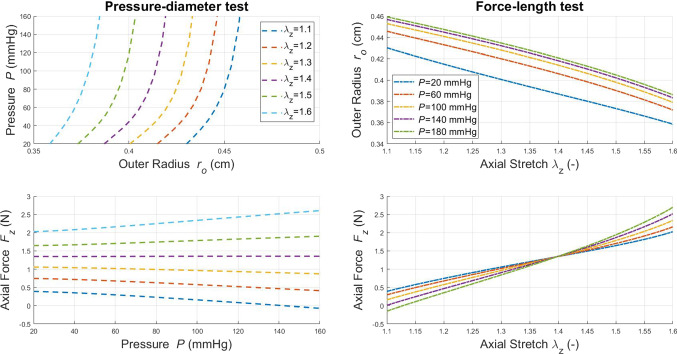
Structural response of hyperelastic wall model under biaxial testing conditions. The panels on the left side are relative to the “Pressure-diameter test”, whilst the panels on the right side are associated with the “Force-length test”. The outer radius in the loaded configuration is defined as $r_o$=$r_i+h$

Here we show how the chosen hyperelastic constitutive model performs under two laboratory tests commonly used for the mechanical characterization of blood vessels. The “Pressure-diameter test” is carried out at fixed vessel length, and thus, the axial stretch $\lambda _z$ is maintained constant. On the other hand, the “Force-length test” is performed at constant internal pressure. These tests are repeated by setting different axial length and inner pressure, respectively. According to Ferruzzi et al. (2013), arteries are known to show, at in-vivo axial stretch, a flat relationship between pressure and axial force. Therefore, the following results can be used for identifying the axial stretch corresponding at *in-vivo* conditions of the vessel. The panels on the left side of Fig. 3 report the numerical results for the “Pressure-diameter test”. In this case the vessel stiffens (pressure-outer radius curve is shifted towards left) for increasing values of axial stretch. At the same time, the axial force exhibits a behaviour with respect to pressure that is strongly dependent on axial stretch. It is important to note that for an axial stretch $\lambda _z$=1.4 the axial force remains unaltered with respect to the inner pressure.

The panels on the right side of Fig. 3 show how outer radius and axial force are affected by axial stretch in a“Force-length test”. For an axial stretch $\lambda _z$=1.4, the axial forces computed for different internal pressures coincide. It is clear that this latter value may provide a good representation of the physiological axial conditions, and therefore, it is adopted in all the simulation cases reported hereafter.

### Effect of tube law approximation

**Fig. 4:**
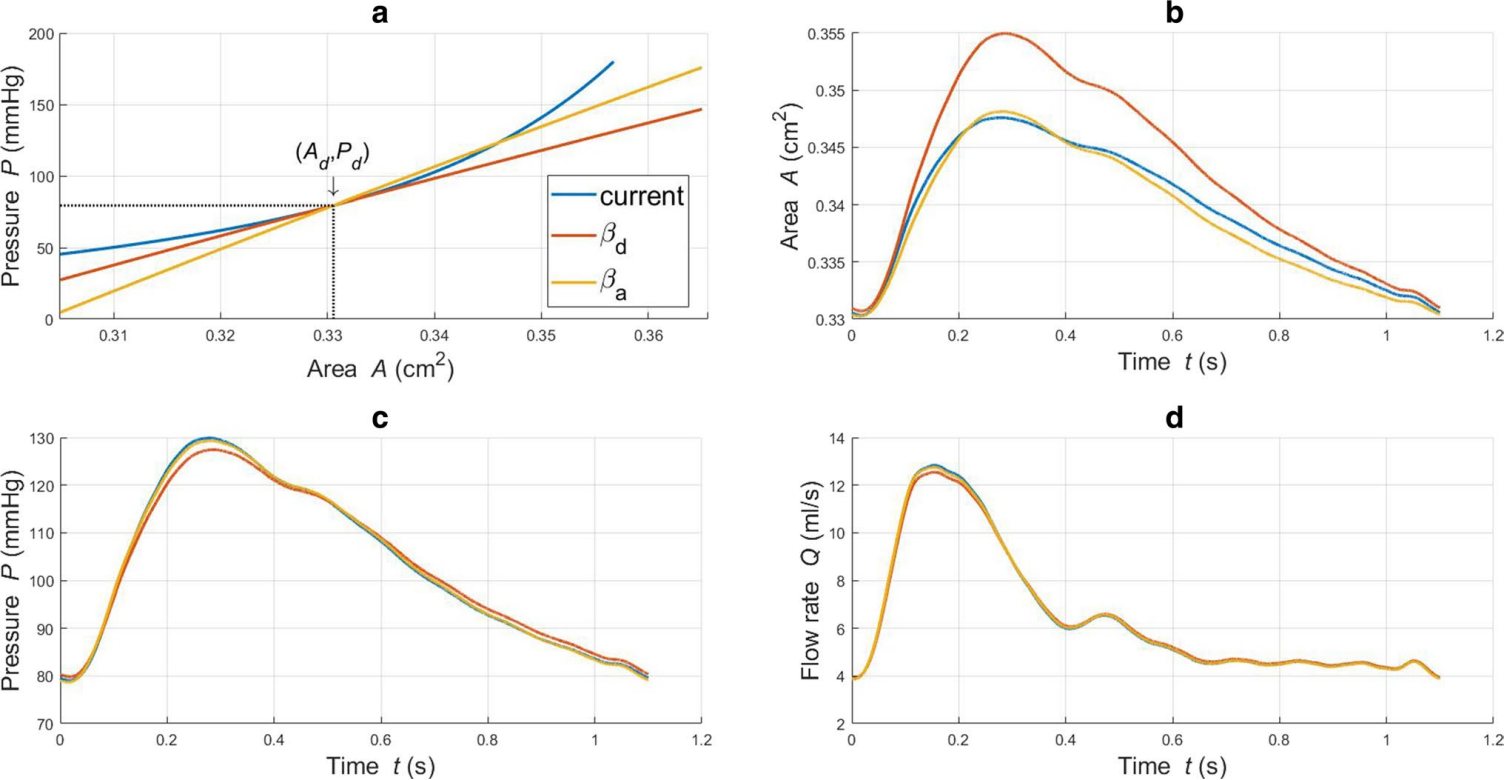
**a**: luminal pressure against cross-sectional area for different tube laws; **b, c, d**: area, pressure and flow waveforms recorded in time at *z*=*l*/2 for different tube laws

**Fig. 5:**
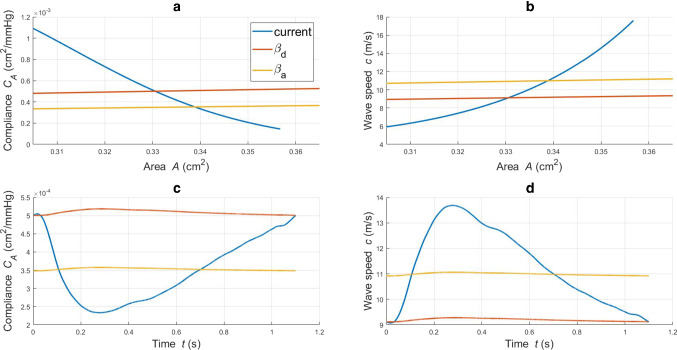
**a, b**: compliance and wave speed against cross-sectional area for different tube laws; **c, d**: compliance and wave speed recorded in time at *z*=*l*/2 for different tube laws

In this section, the effect of the geometric and material non-linearities characterizing the proposed model on the CCA haemodynamics is presented. To this end, a comparison between blood flow and pressure waveforms is carried out across the hyperelastic model and the simpler structural model reported in Sect. 3.3. The simulation results for these three haemodynamic models, for the reference set of parameters, are presented in Figs. 4 and  5. Through Fig. 4a, it is possible to appreciate the *P*-*A* relationship for each structural model. The $\beta _d$ and $\beta _a$ tube laws provide two distinct linearized counterparts for the current model. Figure  4b,c,d shows the values of area, pressure and flow against time recorded at the middle section of the axial length, $z=l/2$. The $\beta _a$ model, by accounting for an averaged compliance, is able to better capture the behaviour of the “non-linear” current approach.

The three models offer significantly different compliance responses for the considered range of cross-sectional area (Fig. 5a). In Fig. 5b, distinct relationships between the intrinsic wave speed and lumen area are delineated. Figure 5c,d shows how the tube law impacts on the vessel compliance and wave speed recorded in time at the central axial location $z=l/2$. Also in this case, the $\beta _a$ approach exhibits a behaviour which better represents the average pattern of the non-linear hyperelastic model. We speculate that the differences between model results may become more pronounced under supra-physiological conditions (for example, in hypertension).

**Fig. 6:**
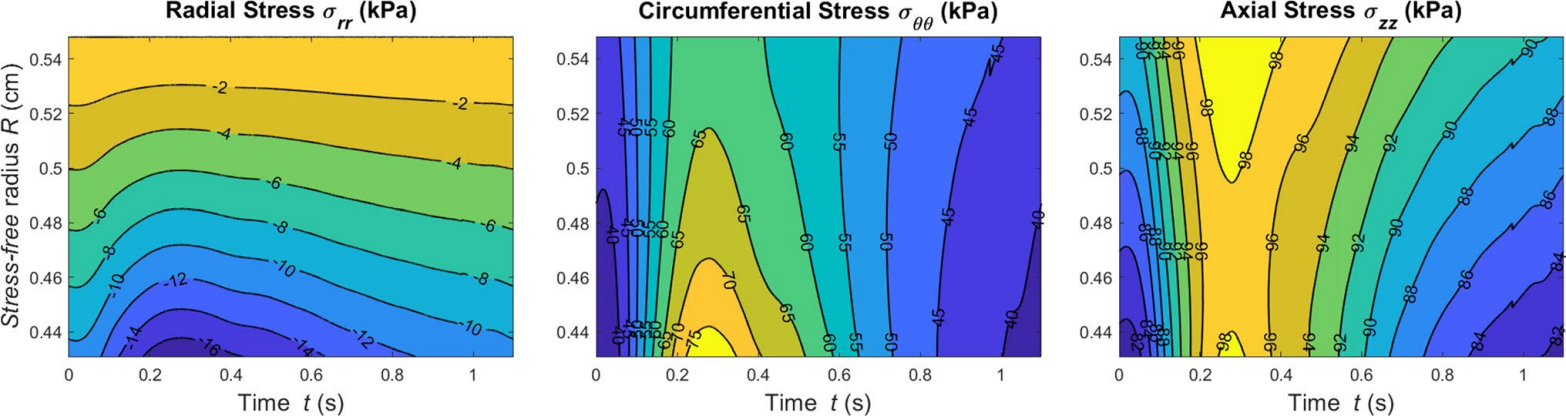
Contour plots of Cauchy stress components against wall thickness and time at *z*=*l*/2

An advantage of the proposed method is that in-tissue variation of the stresses can be computed. Figure 6 shows how the stress along the principal directions varies across the wall thickness and in time at the central axial location $z=l/2$.

Among the wall stress tensor components, the $\sigma _{\theta \theta }$ field seems to experience the largest variation. This information is extremely valuable since it allows a spatial quantification of the periodic mechanical stimuli experienced by the tissue.

### Sensitivity analysis of structural parameters on haemodynamics

Here, the influence of vascular wall parameters on carotid haemodynamics is investigated by carrying out a multivariate sensitivity analysis. The considered output of the CCA model is the local PWV, which is calculated as vessel length divided by the time taken by the foot (diastolic point) of the pressure waveform to travel this distance. Sobol’s sensitivity analysis is carried out by considering the PWV as function of eight input parameters:

28 $$\begin{aligned} {\text{PWV}}={\text{PWV}}(H,R_i,\omega ,k_0,k_1,k_2,\alpha ,\phi ^i). \end{aligned}$$

**Fig. 7:**
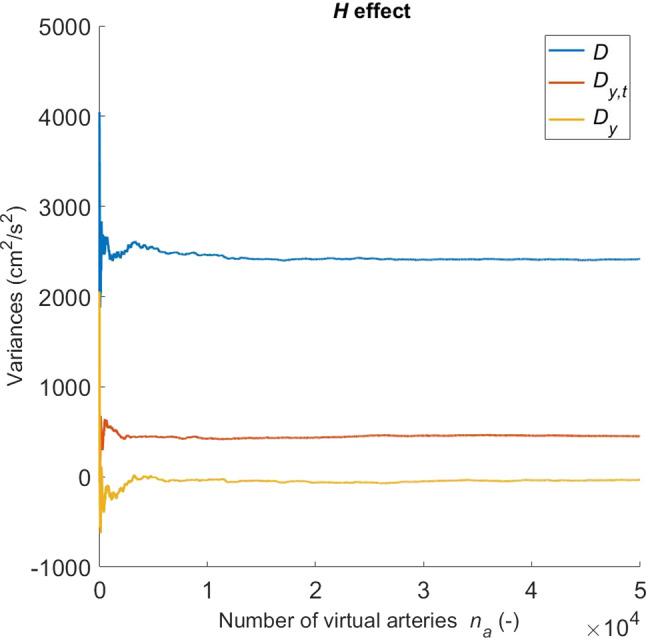
PWV variances associated with *H* variation against number of virtual arteries

Here $\lambda _z$ is kept equal to the physiological value 1.4 (found in Sect. 4.1) and its role on the CCA haemodynamics is investigated in the dedicated Sect. 4.5. For each input parameter *y*, the global sensitivity indices $S_y$ and $S_{y,t}$ are computed by following the methodology reported in the seminal work by Sobol’ (2001). The index $S_y$ is given as ratio between the first order variance due to *y* ($D_y$) and the global variance (*D*), whilst $S_{y,t}$ is defined as total variance due to *y* ($D_{y,t}$) over the global variance. Therefore, $S_y$ represents the effect of only the direct, first order, link between *y* and PWV, whilst $S_{y,t}$ accounts for the total contribution (i.e. the sum of first order and all interactions with remaining parameters) of *y* to PWV. This method requires the definition of two eight-dimensional boxes containing $n_a$ sets of parameters. In the current study these can be seen as $n_a$ “virtual” arteries. These boxes are filled by using two Sobol’s low discrepancy series (via MATLAB Statistics and Machine Learning Toolbox function sobolset) (Sobol’ 1967). Boundary values for these boxes are defined by taking the ± 5 $\%$ variations in the reference values reported in Table 1 (for $\alpha$ the range is 0.95–1.00). The computation of the sensitivity indices requires, for each input parameter, solving four integrals via the Monte Carlo method (see Appendix A2). For this analysis, a sample of $n_a$=50,000 virtual arteries for each box is considered. This ensures all variances converge to a stable solution. See, for example, Fig. 7, which shows how the variances *D*, $D_{y,t}$ and $D_{y}$ change with respect to the sample size for the parameter *H*.

For some input parameters, convergence was reached only for a large samples ($n_a$ >30,000), thus justifying our choice of $n_a$=50,000. We experienced that the oscillatory patterns recorded for some variables may be reduced by increasing the computational precision, i.e. setting lower solver tolerances.

For such a sampling scheme, the computed global variance *D* is 2415.01 cm$^2$/s$^2$, and the indices associated with the parameters of (28) are reported in Table 2.

**Table 2:** Global sensitivity indices for a sample of $n_a$=50,000 virtual arteries obtained by varying the input parameters within a box ± 5$\%$ of data reported in Table 1 corresponding to Sommer’s experiment. $^*$value is rounded to 0.0000 when values are smaller than $10^{-4}$

	*H*	$R_i$	$\omega$	$k_0$	$k_1$	$k_2$	$\alpha$	$\phi ^i$
$S_y$ (= $D_{y}/D$)	0.0000$^*$	0.1446	0.0036	0.0000$^*$	0.0000$^*$	0.0000$^*$	0.0000$^*$	0.0000$^*$
$S_{y,t}$ (= $D_{y,t}/D$)	0.1864	0.9896	0.7693	0.5849	0.4024	0.5647	0.5254	0.6366

The indices reported in Table 2 indicate that $R_i$ plays the most important role in determining local PWV, followed by the other geometric parameter $\omega$. The material constants $\phi ^i$, $k_0$, $k_2$, $\alpha$ and $k_1$ seem to have a more limited impact on the results, especially if the direct/first order contribution is considered. On the other hand, the PWV value is not affected significantly by the choice of the parameter *H*. It is worth stating that these results are valid for a model representing the CCA, and therefore, the parameters’ role may differ for other types of arteries. Nonetheless, the proposed methodology can be readily extended to other artery segments or larger networks of arteries.

Lastly, from the dataset of virtual arteries, we compute the Pearson’s correlation coefficients between the parameters and PWV, and the corresponding linear regression coefficients (Table 3). These reinforce the results of the global sensitivity analysis, i.e. the parameters $R_i$ and $\omega$ are most significant in determining the PWV response.

**Table 3:** Variances of the parameters, correlation with PWV and associated regression coefficients

Parameter	Variance	Std. dev.	Correlation with PWV	Regression Coefficient (slope)
*H* (cm)	1.0 $10^{-5}$	3.4 $10^{-3}$	−0.006	−90.36963
$R_i$ (cm)	1.5 $10^{-4}$	1.2 $10^{-2}$	0.211	833.18354
$\omega$ ($^o$)	5.5 $10^{0}$	2.3 $10^{0}$	−0.079	−1.66925
$k_0$ (kPa)	5.9 $10^{7}$	7.7 $10^{3}$	−0.005	−0.00003
$k_1$ (kPa)	3.6 $10^{7}$	6.0 $10^{3}$	−0.002	−0.00002
$k_2$ (−)	2.7 $10^{0}$	1.6 $10^{0}$	−0.012	−0.35579
$\alpha$ (−)	2.1 $10^{-4}$	1.4 $10^{-2}$	0.023	77.60300
$\phi ^i$ ($^o$)	5.2 $10^{-1}$	7.2 $10^{-1}$	−0.041	−2.79135
PWV (cm/s)	2.4 $10^{3}$	4.9 $10^{1}$	1.000	1.00000

### Volumetric inflow effect

Here the performances of the proposed model are evaluated by considering a perturbation of the volumetric inflow. The reasoning behind this is to simulate changes in physiological state and assess the effect of model (linear vs hyperelastic) choice. In practice, this is relevant because in many cases the model parameters are estimated in one physiological state due to availability of measurements in that state, and yet the models are used to make predictions for a different state. Here, we considered a perturbed state with increased volumetric flow rate (for instance, in hyperaemia). For doing this, differences in the solution between current and $\beta$ models are evaluated under both normal and increased flow conditions. The latter refers to the normal inflow signal increased by 30$\%$. According to the findings of the global sensitivity analysis presented in Sect. 4.3, the values of $R_i$ and $\omega$ significantly affect the structural response of the vessel. Therefore, three distinct virtual arteries are considered: VA1($R_i$=0.3448 cm, $\omega$=64.72$^o$), VA2($R_i$=0.3879 cm, $\omega$=72.81$^o$) and VA3($R_i$=0.4741 cm, $\omega$=88.99$^o$). For each proposed artery, it was verified that the axial stretch $\lambda _z$=1.4 guarantees axial force preservation for varying pressure. All the other parameters are set as in Sect. 4.2. It is important to reiterate that the parameters of all the models remain unchanged between normal and increased flow conditions. Thus, the values of $\beta _d$ and $\beta _a$ are calculated for the normal flow conditions, and they are not recomputed for the increased flow conditions.

**Fig. 8:**
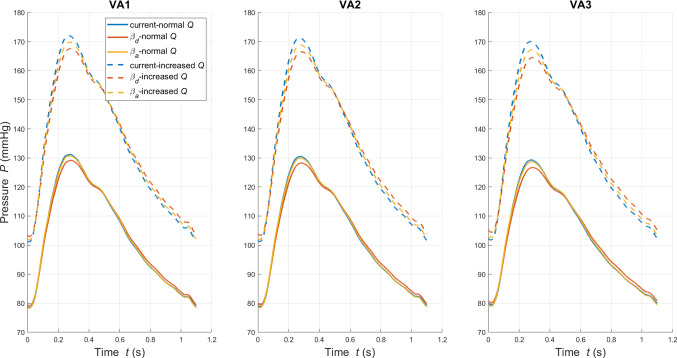
Pressure recorded in time at *z*=*l*/2 for VA1, VA2 and VA3 under normal and increased flow conditions. For each virtual artery, three tube laws are considered

Pressure waveforms for the three arteries under normal and increased flow conditions are reported in Fig. 8. Table 4 shows the haemodynamic variables PWV, $P_s$, $P_d$, $A_s$ and $A_d$ for all cases. As expected, all the monitored variables of VA1, VA2 and VA3 rise with higher flow. The variability in terms of PWV across VA1, VA2 and VA3 is remarkable and confirms the major role of $R_i$ and $\omega$ in the vascular response. With respect to the hyperelastic law, the $\beta _d$ approach seems to provide, for normal flow conditions, closer results in terms of PWV but systolic pressure and area are, respectively, much more underestimated and overestimated than the $\beta _a$ case. In the increased flow case the $\beta _a$ approach provides a better estimation of all quantities. A general trend of increased differences between the pressure waveforms of different models across the two states is also observed.

**Table 4:** Haemodynamic quantities PWV (in m/s), $P_s$ (in mmHg), $P_d$ (in mmHg), $A_s$ (in cm$^2$) and $A_d$ (in cm$^2$) obtained through the current and $\beta$ tube laws for VA1, VA2 and VA3. Values are reported for both normal and increased flow conditions

	Normal *Q*					Increased *Q*				
VA-tube law	PWV	$P_s$	$P_d$	$A_s$	$A_d$	PWV	$P_s$	$P_d$	$A_s$	$A_d$
VA1-current	6.09	131.23	78.57	0.2192	0.2059	7.00	171.98	101.10	0.2248	0.2129
VA1-$\beta _d$	6.12	129.16	79.17	0.2253	0.2061	6.40	167.65	102.98	0.2407	0.2151
VA1-$\beta _a$	6.70	130.72	78.28	0.2197	0.2059	6.89	169.79	101.77	0.2303	0.2120
VA2-current	7.33	130.51	78.78	0.2797	0.2644	8.18	171.26	101.24	0.2863	0.2725
VA2-$\beta _d$	7.37	128.24	79.47	0.2865	0.2647	7.64	166.50	103.38	0.3042	0.2752
VA2-$\beta _a$	7.97	129.97	78.44	0.2802	0.2644	8.13	168.84	102.00	0.2924	0.2715
VA3-current	9.92	129.30	79.41	0.4230	0.4037	11.05	170.05	101.79	0.4319	0.4141
VA3-$\beta _d$	10.08	126.71	80.22	0.4308	0.4041	10.41	164.54	104.38	0.4531	0.4179
VA3-$\beta _a$	10.68	128.69	78.99	0.4235	0.4036	10.96	167.19	102.73	0.4392	0.4130

The percentage error *e* of all haemodynamic quantities between the hyperelastic and the $\beta$ approaches is reported in Table 5. The difference in peak pressure between tube laws is augmented with increasing flow, with the corresponding relative errors presenting a non-negligible increment. The same is observed for the percentage variations in $P_d$, $A_s$ and $A_d$. In some of the cases, the percentage error surprisingly reduces for higher flow. These findings show that the linearized tube laws are able to capture most of the features of the current model’s response, but the accuracy is not preserved across all the haemodynamic variables. An interesting observation is that for both states, the errors for PWV and all other variables show a different behaviour between $\beta _d$ and $\beta _a$ models. Under normal flow conditions, $\beta _d$ produces lower errors for PWV, whilst for all the other variables $\beta _a$ produces a lower error. This suggests that when good accuracy is required for both PWV and the other haemodynamic variables, neither $\beta _a$ nor $\beta _d$ presents an ideal solution.

**Table 5:** Relative variation in PWV, $P_s$, $P_d$, $A_s$ and $A_d$ between the current and $\beta$ tube laws for VA1, VA2 and VA3. Values are reported for both normal and increased flow conditions

VA-$\beta$	Normal *Q*					Increased *Q*				
	*e*(PWV)%	*e*($P_s$)%	*e*($P_d$)%	*e*($A_s$)%	*e*($A_d$)%	*e*(PWV)%	*e*($P_s$)%	*e*($P_d$)%	*e*($A_s$)%	*e*($A_d$)%
VA1-$\beta _d$	0.49	−1.58	0.77	2.78	0.10	−8	−2.52	1.85	7.07	1.03
VA1-$\beta _a$	10.11	−0.38	−0.38	0.23	0.00	−1.64	−1.27	0.66	2.43	−0.44
VA2-$\beta _d$	0.58	−1.74	0.87	2.44	0.11	−6.67	−2.78	2.11	6.23	0.99
VA2-$\beta _a$	8.86	−0.41	−0.44	0.19	−0.01	−0.65	−1.41	0.75	2.14	−0.38
VA3-$\beta _d$	1.60	−2.00	1.03	1.84	0.10	−5.79	−3.24	2.55	4.90	0.90
VA3-$\beta _a$	7.63	−0.47	−0.53	0.12	−0.02	−0.87	−1.68	0.92	1.69	−0.27

**Fig. 9:**
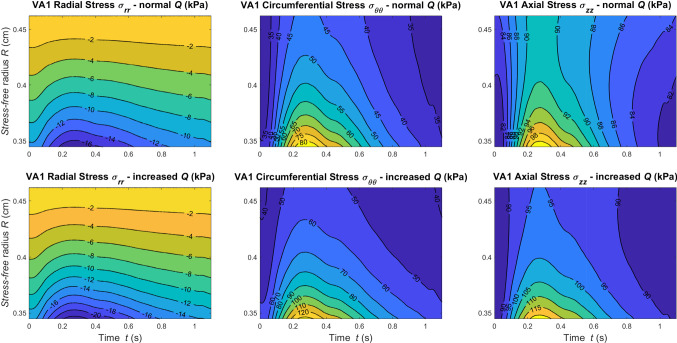
Contour plots of Cauchy Stress components against wall thickness and time at *z*=*l*/2 for VA1 under normal and increased flow conditions

**Fig. 10:**
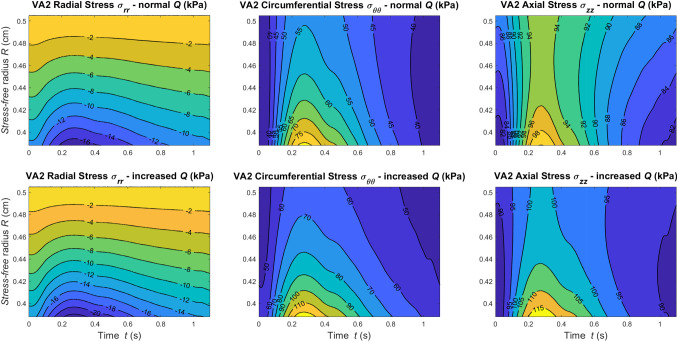
Contour plots of Cauchy Stress components against wall thickness and time at *z*=*l*/2 for VA2 under normal and increased flow conditions

**Fig. 11:**
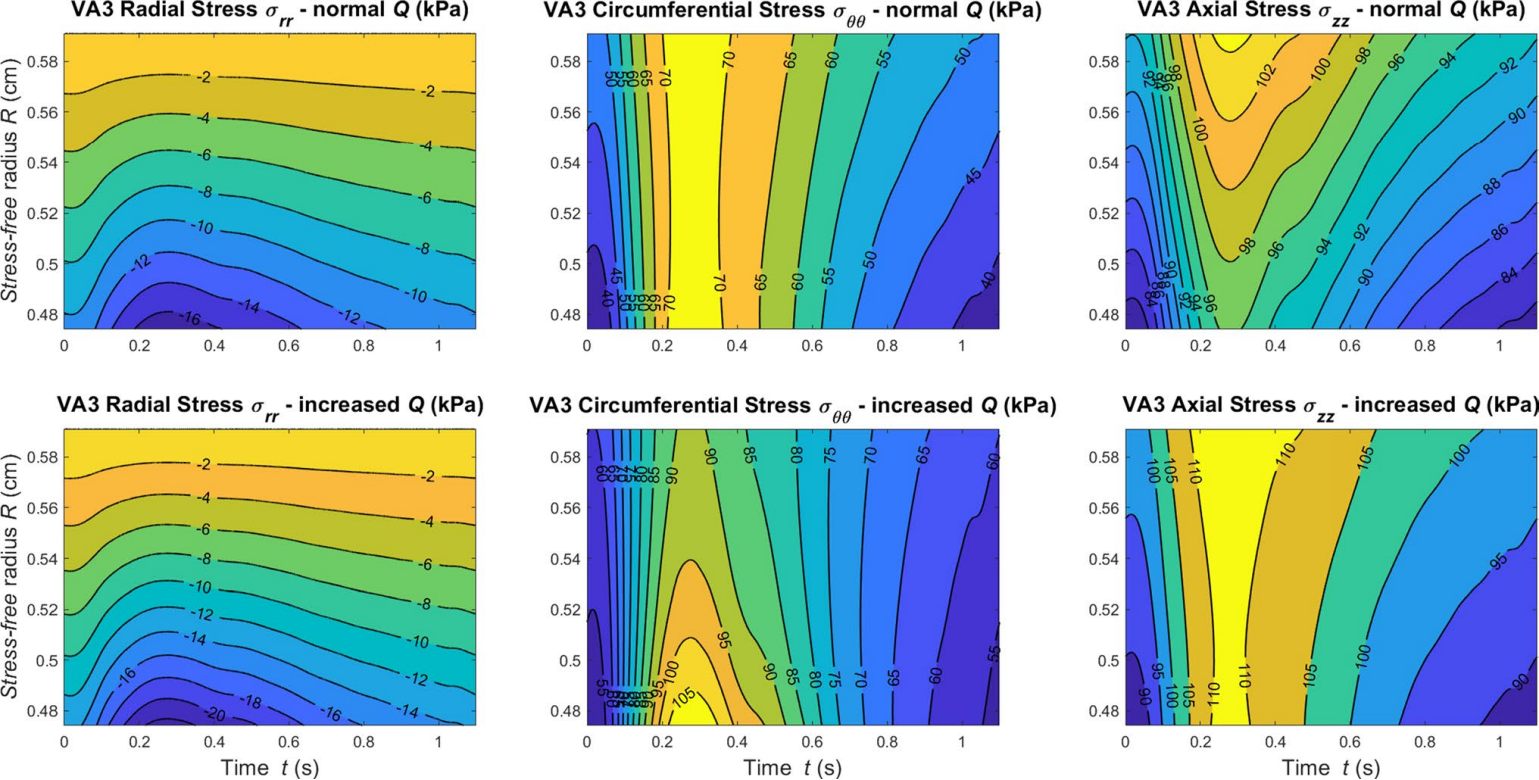
Contour plots of Cauchy Stress components against wall thickness and time at *z*=*l*/2 for VA3 under normal and increased flow conditions

In Figs. 9, 10 and 11, we report the stress along the principal directions across the wall thickness and in time at the central axial location $z=l/2$ of VA1, VA2, and VA3, respectively. Results are reported for both normal and increased flow conditions.

As expected, for higher flow more pronounced stress gradients are recorded. The differences between these results and the stress fields reported in Fig. 6 highlight the strong dependency of the wall structural response on the parameters $R_i$ and $\omega$.

### Influence of axial stretching

**Fig. 12:**
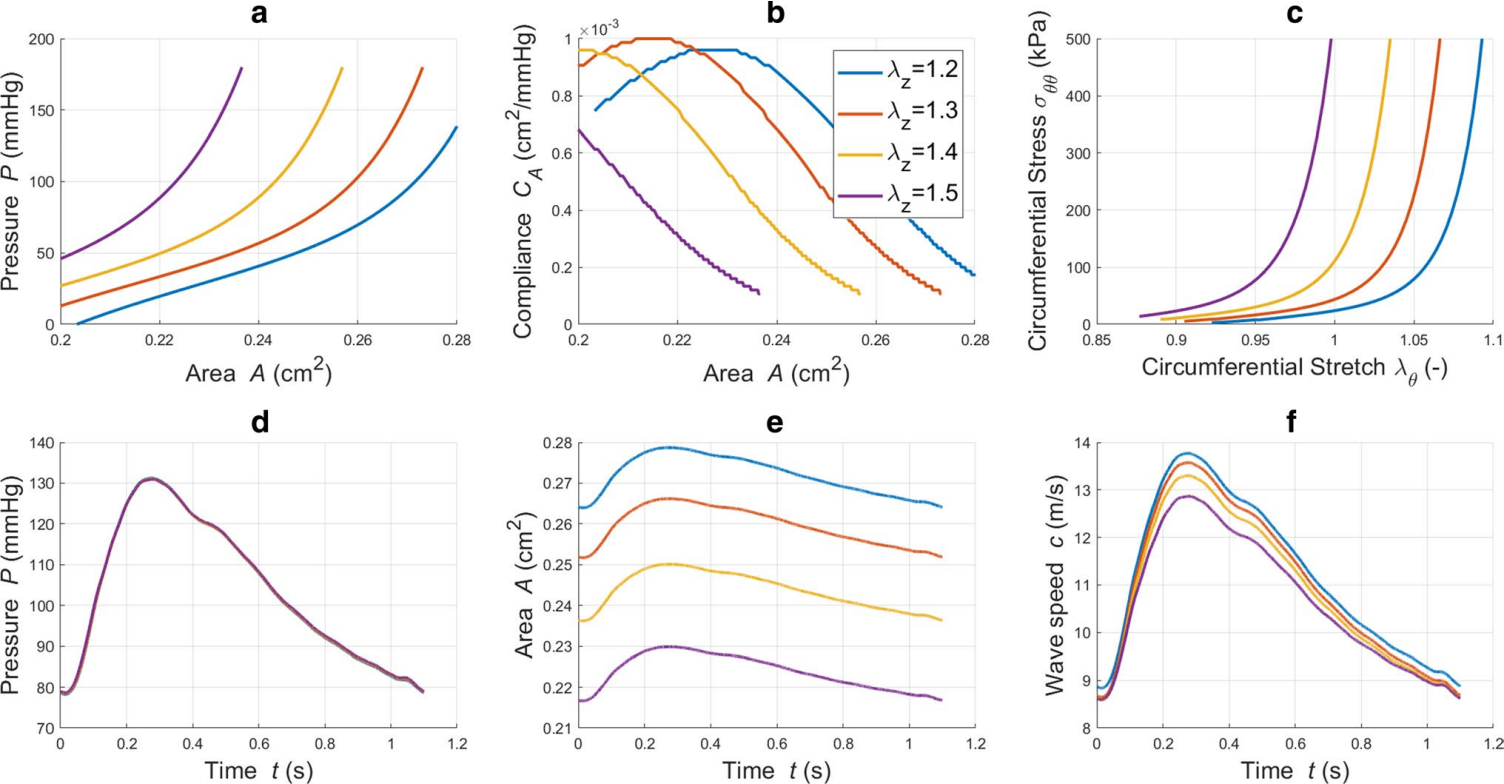
**a, b**: pressure and compliance against cross-sectional area for different axial stretches;** c**: circumferential Cauchy stress component against circumferential stretch at $r_o$ for different axial stretches; **d, e, f**: pressure, area and wave speed recorded in time at *z*=*l*/2 for different axial stretches

As anticipated, arteries are subjected to variable axial loading during their lifespan. The variation in axial forces may occur gradually, as part of a remodelling process, or more suddenly, due to external events such as surgery or impact. In this study, the focus is purely on the haemodynamic variations induced by axial stretching independent from growth or re-adaptation. Here, four axial stretches are considered: $\lambda _{z}$=1.2, 1.3, 1.4 and 1.5. The impact of $\lambda _z$ on the CCA haemodynamics is evaluated by analysing the local PWV, structural capacities and blood waveforms (Fig. 12).

In order to reinforce our analysis, we also considered the vessel distensibility and the associated local ${\text{PWV}}_{HP}$ as metrics for assessing changes in vascular stiffness. Table 6 reports, for each axial extension case, the associated haemodynamic variables.

**Table 6:** Haemodynamic quantities for different total axial stretches. The values of pressure and area are recorded at *z*=*l*/2. $\varDelta P=P_s-P_d$ and $\varDelta A =A_s-A_d$

$\lambda _{z}$	PWV (m/s)	$P_d$ (mmHg)	$\varDelta P$ (mmHg)	$A_d$ (cm$^2$)	$\varDelta A$ (cm$^2$)	$\frac{\varDelta A}{A_d}$ (−)	*D* (mmHg$^{-1}$)	${\text{PWV}}_{HB}$ (m/s)
1.2	9.47	78.71	51.63	0.3689	0.0183	0.0495	9.588 $10^{-4}$	11.45
1.3	9.07	78.93	51.14	0.3519	0.0179	0.0509	9.945 $10^{-4}$	11.25
1.4	8.57	79.07	50.82	0.3303	0.0173	0.0523	10.287 $10^{-4}$	11.06
1.5	8.08	79.16	50.60	0.3031	0.0165	0.0545	10.767 $10^{-4}$	10.81

As shown in Fig. 12a, the “static” relationship linking pressure with area is strongly affected by the axial stretching condition. However, it is important to note that, with axial extension, the shape of *P*-*A* curve is preserved. Similar observations can be made for compliance and circumferential Cauchy stress (Fig. 12b,c). On the contrary, the pressure waveform transiting along the vessel length is not affected by the structure axial extension (Fig. 12d). The same was also observed for the flow waveform. On the contrary, the cross-sectional area waveform is not preserved for variable axial loading conditions. Due to the incompressibility constrain indeed, the more the vessel is axially stretched, the less it can radially deform. This is inline with the area waveforms reported in Fig. 12e. Axial stretching therefore affects the capacity of the vascular wall to deform in the radial direction, without altering the fluid pressure. In addition, Fig. 12f shows that for stretched arteries the intrinsic wave speed of the wall is damped. This is inline with the differences in distensibility and local PWV reported in Table 6. It is important to notice that local PWV evaluated via the Bramwell–Hill ($\text {PWV}_{HP}$) provides higher values than the reference one. These findings are partially in agreement with what experimentally found by Holtackers et al. (2016). In such experimental study, both absolute and relative vessel distension (calculated by using diameters instead of areas) slightly decreased from the normal to the stretched case. In our case, only the absolute difference in area $\varDelta A$ reduced for higher $\lambda _z$. We believe that this might be due to either the diverse system’s geometry (vessel size and pre-stress conditions) or to the choice of constitutive law. Indeed, for the considered vessel’s settings, the physiological range of circumferential stress (and of pressure) corresponds to circumferential stretch $\lambda _{\theta }$ $\le$ 1.1, which is qualitatively similar to the results reported in Sommer and Holzapfel (2012) (see Figure 10 therein). Although in the work by Spronck and Humphrey (2019) (see Figure 2 therein) the vessel appears much more stretched along the circumferential direction (1.0$\le$ $\lambda _{\theta }$ $\le$ 1.7), the response of the wall, in terms of circumferential stress, to axial stretching is in line with our results. Therefore, our results might indicate that circumferential compression condition might limit the decrease in distension with axial stretching. It is important to remark that such topic deserves further investigation. These findings may be important for the study of molecular mechanisms which are affected or regulated by vessel stretches such as growth and remodelling.

## Conclusions

In this study, we introduced a novel framework for realistically characterizing the intricate relationship between the vascular wall and blood flow in the human CCA. The presented computational model integrates a comprehensive representation of the arterial structure into a one-dimensional blood haemodynamics solver. The effect of non-linearities introduced with the proposed model on haemodynamic results was firstly investigated by using a popular “linearized” tube law for the comparison. In this respect, substantial differences were recorded for area, compliance and wave speed. Furthermore, it was shown that the “linear” model may represent a low-computational cost surrogate for the current model, as long as the average compliance behaviour is preserved. It is worth noting that the average compliance can be computed from the nonlinear model and then used in current linear solvers without significant modification of existing codes. We also show that, under normal flow conditions, the compliance obtained by linearizing the hyperelastic at diastole produces lower errors for PWV, but for all the other variables (pressure, flowrate, and area) the average compliance model results in lower errors.

We used the PWV as index for characterizing the haemodynamic variations within the system. This quantity is a good indicator of how the information propagates through the vascular system. The sensitivity analysis shed the light on the main structural factors affecting the CCA blood dynamics. In this case, the size and residual stress component seem to have a huge role in the blood–vascular wall interaction. More specifically, the parameters $R_i$ and $\omega$ were associated with a global sensitivity index $S_{y,t}$ > 75 $\%$. On the contrary, the parameter *H* plays a limited effect on the CCA haemodynamics, since it was associated with a global sensitivity index $S_{y,t}$ < 20 $\%$. These findings may benefit the design of future studies by having identified the most important model’s parameters which need to be estimated reliably. However, different conclusions might be drawn if, in the sensitivity analysis, other haemodynamic quantities were considered. In this respect, future studies need to be carried out for investigating which effects can be obtained in different types of arteries and at systemic level. Subsequently, we quantified the inflow effect on the differences between tube laws for three different virtual arteries. These findings further reinforce what has been found in the previous sections and show an unexpected model insensitivity to flow. We believe that the optimal choice among the presented tube laws depends on the type of information sought. For determining a change of physiological state, if absolute errors are important, the hyperelastic model should be preferred (provided a good estimate of parameters is available). In the estimation of PWV for instance, variation in a few percent matters and, therefore, the current model represents a more specific and complete tool for linking PWV with the wall features. On the contrary, if only pressure and flow fields are needed, the linear model should be sufficient. Among the linearized models, $\beta _{a}$ seems to capture better most of the haemodynamic quantities computed via the non-linear model, due its capacity to reflect the averaged wall structural response. On the other hand, the $\beta _{d}$ approach appears superior in the PWV estimation if normal flow state is assumed. During the travel of the pulse pressure, the vessel average compliance is much closer to the compliance at diastole rather than to the averaged compliance value. Ultimately, we documented how axial loading may affect the carotid haemodynamics. This analysis showed that, interestingly, neither blood pressure nor flow are directly affected by axial extension. However, the capacity of the vessel to deform and the pulse transmissivity is significantly compromised. Coming to a conclusion, axial stretching preserves the pressure and flow the waveforms, but it reduces their travelling speed. It is therefore speculated that local PWV may also be informative about the stretching level of arteries. Given the partial discrepancy with some clinical observations, the underlying link between vessel distensibility and geometry under axial loading requires further investigation. Although the analysis exclusively involved the common carotid artery, we believe that these findings might be representative, to some extent, also for other large conduit arteries, such as iliac and brachial artery.

We conclude with the following considerations. It is undeniable that the current framework introduces a higher complexity and number of parameters with respect to the standard *P*-*A* tube laws. However, the advantages for employing the proposed physics-driven model are manifold. In the first place, such wall description allows to represent various types of conditions and component effects, such as axial loading, fibre dispersion, residual stress and potentially, in the future, also the active contractility of the wall. Secondly, the adopted structural model developed by Holzapfel and co-workers was defined for capturing the characteristic non-linear behaviour of the vascular tissue. This aspect becomes extremely relevant every time the vessel is exposed to blood pressure loads which are significantly above the physiological range. Additionally, the presented framework permits to compute the wall stress and strain distribution in a realistic blood dynamic context, giving an inestimable information for characterizing the arterial mechanobiology. We reported a methodology derived in a general form, and therefore, many other strain energy functions can be plugged in. It is worth reiterating that the proposed CCA model can be easily extended to other arterial segments and larger vessel networks.

## Appendix

### A1: Model boundary conditions

Here the details concerning the boundary conditions imposed in the CCA model are reported. The inlet flow rate is obtained via spline interpolation of the digitized waveform from Figueroa et al. (2006):

29 $$\begin{aligned} \begin{aligned} Q_{in}(t)&=6.5+3.294\sin (2\pi t/T-0.023974)\\&+1.9262\sin (4\pi t/T-1.1801)\\&-1.4219\sin (6\pi t/T+0.92701)\\&-0.66627\sin (8\pi t/T-0.24118)\\&-0.33933\sin (10\pi t/T-0.27471)\\&-0.37914\sin (12\pi t/T-1.0557)\\&+0.22396\sin (14\pi t/T+1.22)\\&+0.1507\sin (16\pi t/T+1.0984)\\&+0.18735\sin (18\pi t/T+0.067483)\\&+0.038625\sin (20\pi t/T+0.22262)\\&+0.012643\sin (22\pi t/T-0.10093)\\&-0.0042453\sin (24\pi t/T-1.1044)\\&-0.012781\sin (26\pi t/T-1.3739)\\&+0.014805\sin (28\pi t/T+1.2797)\\&+0.012249\sin (30\pi t/T+0.80827)\\&+0.0076502\sin (32\pi t/T+0.40757)\\&+0.0030692\sin (34\pi t/T+0.195)\\&-0.0012271\sin (36\pi t/T-1.1371)\\&-0.0042581\sin (38\pi t/T-0.92102)\\&-0.0069785\sin (40\pi t/T-1.2364)\\&+0.0085652\sin (42\pi t/T+1.4539)\\&+0.0081881\sin (44\pi t/T+0.89599)\\&+0.0056549\sin (46\pi t/T+0.17623)\\&+0.0026358\sin (48\pi t/T-1.3003)\\&-0.0050868\sin (50\pi t/T-0.011056)\\&-0.0085829\sin (52\pi t/T-0.86463)~~~{(\text {ml/s})}, \end{aligned} \end{aligned}$$

where *T* is set equal to 1.1 s. According to Xiao et al. (2014), the Windkessel resistance $R_1$, compliance *C* and resistance $R_2$ are set equal to 2.4875 $\cdot$ 10$^8$ Pa s/m$^3$, 1.7529 $\cdot$ 10$^{-10}$ m$^3$/Pa and 1.8697 $\cdot$ 10$^9$ Pa s/m$^3$, respectively.

### A2: Sensitivity analysis indices

Here we resume the methodology adopted from Sobol’ (2001) for calculating the global sensitivity indexes $S_y$ and $S_{y,tot}$ for the input parameter *y* of a function *f* (in our study *f*=PWV), which must be integrable over a domain $I^n$. Such function can be defined as $f=f(y,z)$ where *z* are the complementary input parameters of *y*. Consider two independent random points $\xi _j$ and $\xi _j'$ uniformly distributed in $I^n$ and let $\xi$=($\eta$,$\zeta$) and $\xi '$=($\eta '$,$\zeta '$). Given *N* trials (in our study *N*=$n_a$), the variances associated with the input parameters *y* and *z* are calculated by means of Monte Carlo method as

30 $$\begin{aligned} {\left\{ \begin{array}{ll} f_0=\frac{1}{N}\sum _{j=1}^{N} f(\xi _j) , \\ D=\frac{1}{N}\sum _{j=1}^{N} f^2(\xi _j)-f_0^2,\\ D_y=\frac{1}{N}\sum _{j=1}^{N}f(\xi _j)f(\eta _j,\zeta _j')-f_0^2,\\ D_z=\frac{1}{N}\sum _{j=1}^{N} f(\xi _j)f(\eta _j',\zeta _j)-f_0^2, \end{array}\right. } \end{aligned}$$

where $f_0$ is the mean value. The sensitivity indices can be obtained as:

31 $$\begin{aligned} S_y=\frac{D_y}{D}~~~\text {and}~~~~S_{y,t}=1-\frac{D_z}{D}. \end{aligned}$$

### A3: Equilibrium equations in cylindrical coordinates

The momentum balance equations for Cauchy stress in cylindrical coordinates are:

32 $$\begin{aligned} \sigma _{rr,r} + \frac{\sigma _{r\theta ,\theta }}{r} + \sigma _{rz,z} + \frac{\sigma _{rr}-\sigma _{\theta \theta }}{r}&= 0, \end{aligned}$$

33 $$\begin{aligned} \sigma _{r\theta ,r} + \frac{\sigma _{\theta \theta ,\theta }}{r} + \sigma _{z\theta ,z} + \frac{2}{r} \sigma _{r\theta }&= 0, \text { and} \end{aligned}$$

34 $$\begin{aligned} \sigma _{rz,r} + \frac{\sigma _{z\theta ,\theta }}{r} + \sigma _{zz,z} + \frac{\sigma _{rz}}{r}&= 0, \end{aligned}$$

in the radial, circumferential and axial directions, respectively. By axisymmetry, all the derivatives with respect to $\theta$ are zero. Given the stress state (10), all shear stresses are also zero. Therefore, the radial direction momentum balance gives equation (11), the circumferential direction momentum balance is identically satisfied, and the axial direction momentum balance gives $\sigma _{zz,z}=0$. From equation (11), radial stress at any radius *r* can be written as

35 $$\begin{aligned} \sigma _{rr}(r) = -P_{in} + \int \limits _{r_i}^{r} \frac{\sigma _{\theta \theta }-\sigma _{rr}}{r} dr, \end{aligned}$$

and, therefore, the incompressibility Lagrange multiplier at radius *r* can be written as:

36 $$\begin{aligned} p(r)&= -\lambda _r\frac{\partial \varPsi }{\partial \lambda _r} + \sigma _{rr}= -\lambda _r\frac{\partial \varPsi }{\partial \lambda _r} -P_{in} + \int \limits _{r_i}^{r} \left( \lambda _{\theta }\frac{\partial \varPsi }{\partial \lambda _{\theta }}-\lambda _{r}\frac{\partial \varPsi }{\partial \lambda _{r}}\right) \frac{1}{r}dr. \end{aligned}$$

The axial force required for the applied axial stretch $\lambda _z$ is balanced by $\sigma _{zz}$ and the internal pressure on the ends, i.e.

37 $$\begin{aligned} F_z&= \int \limits _{r_i}^{r_i+h} 2\pi r \sigma _{zz} dr - \pi r_i^2 P_{in}= \int \limits _{r_i}^{r_i+h} 2\pi r \left( \lambda _{z}\frac{\partial \varPsi }{\partial \lambda _{z}}-p\right) dr - \pi r_i^2 P_{in}. \end{aligned}$$

Substituting *p*(*r*) from equation (36) in equation (37) and reversing the order of integration (Holzapfel et al. 2000) give equation (15).
